# Analysis of volatile compounds and flavor fingerprint in hairtail (*Trichiurus lepturus)* during air-drying using headspace-gas chromatography-ion mobility spectrometry (HS-GC-IMS)

**DOI:** 10.3389/fnut.2022.1088128

**Published:** 2023-01-11

**Authors:** Yueqin Liao, Yixuan Ding, Yingru Wu, Qi Du, Jiangyue Xia, Junqi Jia, Huimin Lin, Soottawat Benjakul, Bin Zhang, Yi Hu

**Affiliations:** ^1^Key Laboratory of Health Risk Factors for Seafood of Zhejiang Province, College of Food Science and Pharmacy, Zhejiang Ocean University, Zhoushan, China; ^2^Pisa Marine Graduate School, Zhejiang Ocean University, Zhoushan, China; ^3^Faculty of Agro-Industry, International Center of Excellence in Seafood Science and Innovation, Prince of Songkla University, Hat Yai, Thailand

**Keywords:** air-drying, hairtail, HS-GC-IMS, volatile flavors, fingerprint

## Abstract

In the present study, changes in volatile compounds during processing were analyzed using the headspace-gas chromatography-ion mobility spectrometry (HS-GC-IMS), to investigate the generation of aroma in hairtails (*Trichiurus lepturus*) during air-drying. Physicochemical indices, such as moisture content and thiobarbituric acid reactive substances (TBARS), were also detected. Flavor fingerprints were studied and developed to distinguish the samples of fresh hairtails (0 day) from air-dried hairtails (2 and 4 days). A total of 75 volatile organic compounds (VOCs) were identified in hairtails, in which alcohols, aldehydes, ketones, and esters were the principal contributors to the formation of the overall flavor of hairtails during air-drying. Seven flavor compounds (ethanol, 3-methyl-1-butanol, 1-pentanol, hexanal, octanal, benzaldehyde, and 3-methylbutanal), two flavor compounds (acetoin and dimethyl sulfide), and eight flavor compounds (1-hexanol, 1-octen-3-ol, nonanal, heptanal, 2-heptanone, ethyl acetate, trimethylamine, and ammonia) were identified in 0, 2, and 4 air-dried hairtails as biomarkers, respectively. The results showed that HS-GC-IMS could detect VOCs in different air-dried hairtails rapidly and comprehensively.

## 1. Introduction

Hairtail (*Trichiurus lepturus*) is one of the most economic marine fish in China and is mainly distributed in the Eastern Pacific Ocean (1). Air-dried hairtails are one of the most popularly consumed dried seafoods in coastal regions and are of high nutritional value. Compared with the taste and texture of fish products, the volatile flavors of hairtails produce distinctive quality characteristics and have an enormous impact on the evaluation of the freshness and nutritional value of foods (2). Non-enzymatic reactions taking place in the formation of flavor compounds during food processing include Maillard reactions, caramelization reactions, oxidative and thermal degradation of lipids, and degradation of proteins and vitamins. The Maillard reaction, lipid degradation, and a combination of both these reactions are particularly important in flavor formation during processing (3). Most of the flavor components in dry-cured meat products are formed by fat oxidation (4, 5). In addition, the majority of these substances are unstable and form other stable substances. Moreover, chemical reactions mediated by enzymes and microorganisms continue to form unfavorable volatile compounds, in turn affecting product quality (6, 7). Recently, Ding et al. (8) described physicochemical indices and flavor profiles in traditional deep-fried and circulating air-fried hairtails based on headspace-gas chromatography-ion mobility spectrometry (HS-GC-IMS). Significant differences in flavor composition have been reported in traditional deep-fried hairtails compared with circulating air-fried hairtails. Silver carp (*Hypophthalmichthys molitrix*) is a highly nutritious fish from China, India, and Mexico. A recent study compared the lipid oxidation, flavor, and content of fat in silver carp using different drying methods (9). Leduc et al. (10) identified thiophene, hexanal, 1-octen-3-one, dimethyltrisulfide, and 1-nonen-3-ol as potential markers of quality in European seabass (*Dicentrarchus labrax*). However, studies on air-dried hairtails are limited, and changes in hairtail flavor during air-drying have not been investigated to date. Therefore, a simple method to recognize compounds and changes in the flavors of hairtails during the air-drying process is necessary.

The HS-GC-IMS is a gas-phase separation technology that relies on gas chromatography to preseparate samples, and then, the compounds are characterized subsequently based on gas-phase ion mobility (11). IMS is an easy-to-use technique that is inexpensive, shows ultrahigh sensitivity, and does not require time-consuming pretreatment of samples (12). IMS has been used in various areas such as environmental analysis (13), medical analysis (14), and food flavor analysis (12). Wang et al. (15) used GC-IMS to analyze volatile components and flavor fingerprints of samples from Jingyuan lambs of different ages, and they found that alcohols, ketones, aldehydes, esters, and thiazoles show high-intensity peaks in spectrometry. Jia et al. (6) identified volatile organic compounds (VOCs) in chill-stored silver carp by GC-IMS and evaluated changes in VOCs that are induced by three predominant bacteria in chill-stored silver carp after 14 days of storage. Yao et al. (16) investigated the flavor profile of five different regional Chinese smoked chickens through GC-IMS and identified 34 flavor compounds, with a majority of them consisting of heptanal, n-nonanal, furfurol, and hexanal. Nie et al. (17) reported that aldehydes are the principal contributors to the formation of the overall flavor of fermented sea bass based on GC-IMS and gas chromatography-mass spectrometry (GC-MS) analysis.

The present study aimed to identify VOCs in hairtails during the air-drying process using HS-GC-IMS. Principal component analysis (PCA) was performed to assess the efficiency of HS-GC-IMS in the detection and identification of hairtail samples during the air-drying process. The present study also intended to analyze the flavor characteristics of hairtails during the air-drying process to provide theoretical support to processing and quality control in the production of air-dried hairtails.

## 2. Material and methods

### 2.1. Hairtail samples and treatment

Nine fresh individual fish samples belonging to hairtail (*T. lepturus*) species weighing 300–400 g and having a length of 50–60 cm were purchased from a local fish market in Zhoushan, Zhejiang Province, China. Hairtail samples were immersed in flaked ice in a foam box and transported to the laboratory within 30 min. Upon arrival, the fish samples were taken out from the foam box and prepared manually by removing the gills, the viscera, the tail, and the black membrane in the abdominal cavity but they were not sectioned nor the intramuscular bone removed. Another six hairtail individuals were salted with 2% (w/w) sodium chloride (NaCl) and then air-dried under outdoor natural conditions (temperature: 4–7°C, relative humidity: 40–60%) and wind speed: 2–6 m/s). The muscle tissue located below the abdominal region (6.0 × 5.0 × 1.0-cm) from each hairtail was collected for analysis.

### 2.2. Moisture content and thiobarbituric acid reactive substance (TBARS) content analysis

The moisture content of hairtails during the air-drying process was measured according to the method of Ma et al. (18) with minor modifications. Briefly, 2 g of minced samples was placed in a dry container and dried at 105°C. The moisture content of the samples was calculated using the following formula: Moisture content (%) = (*M*_1_ –* M*_2_)/*M*_1_^*^100%, where *M*_1_ and *M*_2_ signify sample weights before and after drying, respectively. The TBARS content of the samples was measured, as described by Wang et al. (19) with minor modifications. Five grams of muscle and 50 mL of 7.5% (v/v) trichloroacetic acid (TCA) solution were added into a centrifuge tube for homogenization in ice for 30 s, followed by centrifugation for 5 min (10,000 × g, 4°C). The supernatant solution (5 mL) and thiobarbituric acid (TBA) solution (0.02 mol/L, 5 mL) were mixed and boiled for 30 min. Finally, the solution was cooled in ice water for 20 min, and the absorbance was measured at a wavelength of 532 nm. The results were expressed as milligrams (mg) of malondialdehyde (MDA) per kilogram (kg) (mg MDA/kg) of muscle.

### 2.3. HS-GC-IMS analysis

Changes in the volatile compounds of hairtails were investigated using HS-GC-IMS (FlavourSpec^®^, G.A.S., Dortmund, Germany) with a wax capillary column (30 m × 0.53 mm × 1 μm) (Restek, USA). Two grams of fish muscle was transferred to a 20-mL headspace vial and incubated at 60°C for 20 min. Then, a headspace-gas sample of 500 μL was automatically drawn by a syringe needle (85°C) and injected into the GC injector (column temperature, 60°C; drift tube temperature, 45°C). High-purity nitrogen was employed as sample gas at a flow rate of 150 mL/min. The carrier gas flow rate was set to 2 mL/min for the first 2 min, at which point the carrier gas flow rate was increased to 10 mL/min for 10 min, to 100 mL/min for 20 min, to 100 mL/min for a 30 min, and then the flow was stopped. The retention index (RI) was calculated with reference to n-ketone C4–C9 (Sinopharm Chemical Reagent Beijing Co., Ltd., China). The VOCs were identified by comparing the RI and the drift time of the standard in the GC-IMS library.

### 2.4. Statistical analysis

All tests were repeated three times. The HS-GC-IMS chromatograms were analyzed by HS-GC-IMS Library Search, Reporter Gallery plot, Dynamic PCA, and Laboratory Analytical Viewer (LAV) processing software (FlavourSpec^®^, G.A.S., Dortmund, Germany). The data were processed further by one-way analysis of variance (ANOVA) using SPSS 21.0 software (SPSS Inc., Chicago, IL, USA), with the significance level set as a *P*-value of < 0.05. Correlation analysis among volatile compounds was performed using Origin 2021 software (Origin-Lab Corporation, Northampton, MA, USA).

## 3. Results and discussion

### 3.1. Moisture content and TBARS content analysis of hairtails during the air-drying process

**Figure 1:**
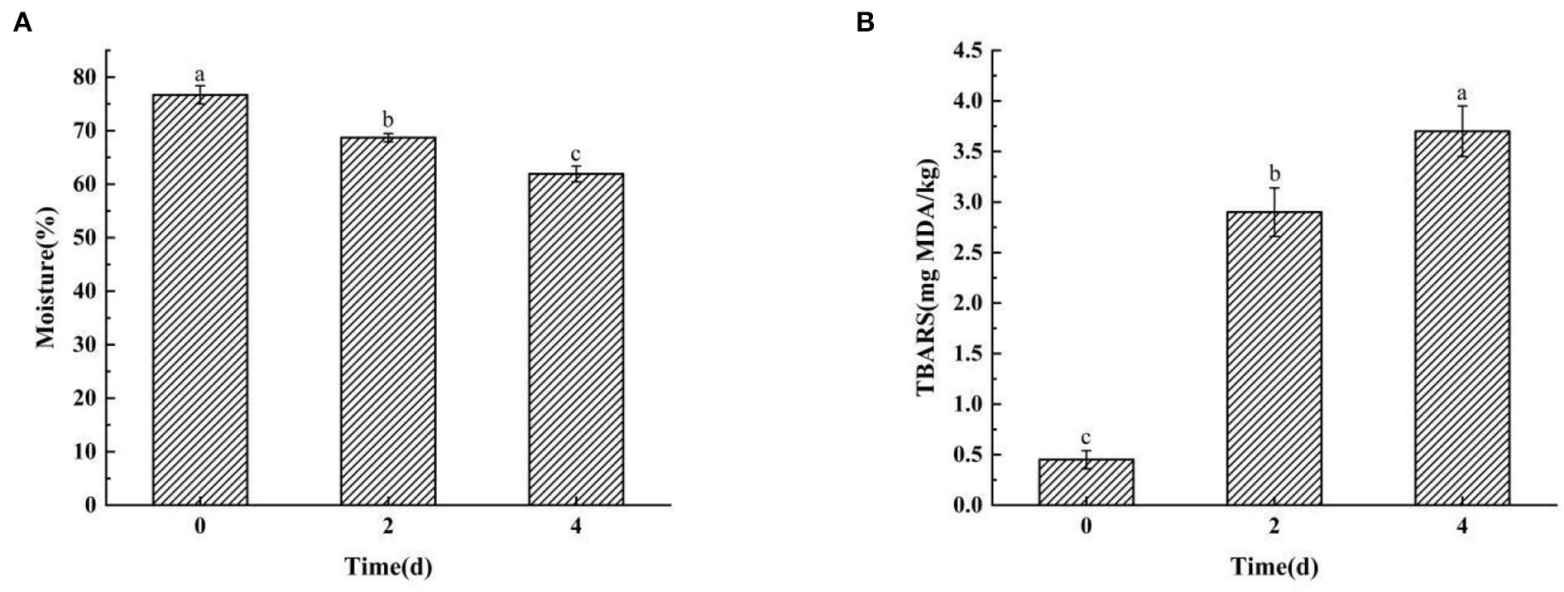
The moisture content **(A)** and thiobarbituric acid reactive substance (TBARS) values **(B)** in hairtails during the air-drying process. Different letters mean each substrate's significant difference (P < 0.05).

The moisture content decreased significantly from 76.69% in raw fish to 61.92% in the final product (*P* < 0.05) with prolongation of air-drying duration (Figure 1A). The results indicated that the air-drying time and salt-drying stage were the major factors that led to water loss in hairtails. Some studies indicated that the generation of ester and 1-octen-3-ol is correlated to a decrease in moisture content (20, 21), which is similar to the present results. Lipid oxidation is recognized as a biochemical reaction that is strongly associated with the production of odor (22). The TBARS values represent the content of aldehydes and ketones that stem from lipid degradation (23). Moreover, the TBARS values increased significantly (*P* < 0.05) with prolongation of air-dried time (Figure 1B), which showed that lipid oxidation occurred rapidly in the hairtail muscle. This feature could be ascribed to the generation of several unstable peroxides by the oxidation and decomposition of unsaturated fatty acids. The peroxide compounds were then converted further into aldehydes, ketones, alcohols, and other shorter-chain hydrocarbons (24, 25). However, the moisture content and TBARS values of hairtails may also be affected by the penetration of salt.

### 3.2. GC-IMS topographic plots in hairtails during air-drying

**Figure 2:**
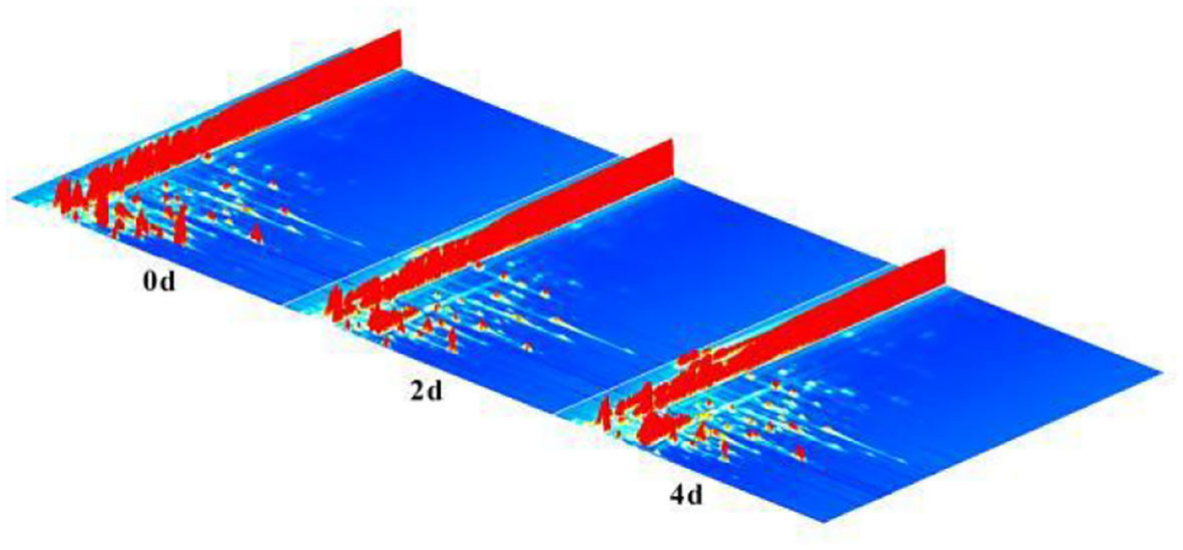
Three-dimensional (3D) topographic plot of hairtails.

The samples of fresh hairtails (0 d), hairtails air-dried for 2 days (2 d), and hairtails air-dried for four days (4 d) were analyzed by GC-IMS to obtain information on volatile compounds (Figure 2). The x-axis, y-axis, and z-axis, respectively, represent the drift time, the gas chromatography retention time, and the signal intensity of ionic compounds. In Figure 2, each point represents a volatile compound, and one compound may have two or three spots representing dimers or multimers (26). These results indicated that the signal peak intensities differ, and the content of volatile compounds also varied among samples.

**Figure 3:**
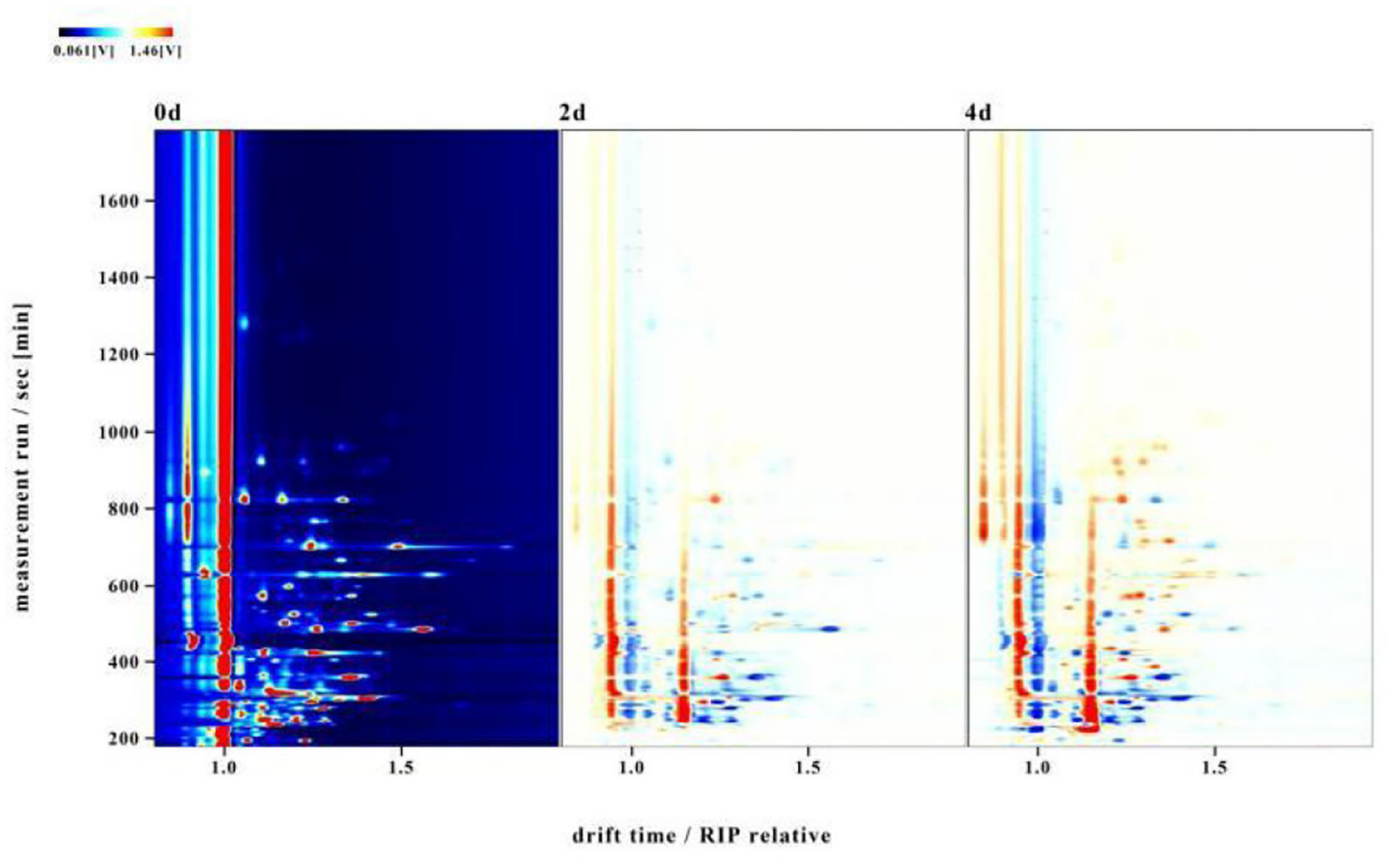
Two-dimensional (2D) spectra of hairtails.

To distinguish differences among volatile compounds in hairtails during the air-drying process, the three-dimensional (3D) topographic plots in Figure 2 were converted into two-dimensional (2D) spectra plots (Figure 3). Differences in air-dried hairtail samples were compared using the difference comparison model. The 2D spectra plot of the 0-day hairtail was used as the reference, and the topographic plots of the other two hairtail samples were obtained from the reference. If the volatile components in different samples were consistent with each other, then the background after subtracting was white, the blue corresponds to the low peak intensity of volatile compounds, and the red to the higher peak intensity of volatile compounds. The results showed that numerous signal peaks appeared in the retention time of 200–800 s and the drift time of 1.0–1.5 s. Compared with 0-day hairtail samples, the 2-day hairtail samples have more blue spots within the range of 200–500 s retention time, indicating that most signal peaks of volatile compounds decreased during early air-drying, and the 4-day hairtail samples have more red spots within the range of 400–1,000 s retention time, indicating that most signal peaks of volatile compounds increased during the later stage of air-drying.

### 3.3. Volatile organic compound identification in hairtails during the air-drying process

The VOCs of hairtail samples were confirmed, according to the drift time, retention time, and retention index of VOCs, and these parameters were then compared with similar parameters cited in the IMS database. Each number represents one VOC, and the results of the identification of each VOC are shown in Table 1. The concentrations of some single VOCs were different and thus produced multiple signals. Ninety signal peaks and 75 VOCs were identified in three hairtail samples compared with the GC-IMS library, including 22 species of alcohols, 21 species of aldehydes, 11 species of ketones, 7 species of esters, 4 species of hydrocarbons, 3 species of nitrogen-bearing, 2 species of pyrazine, 2 species of acid, 1 species of furan, 1 species of sulfur-bearing, and 1 species of pyridine. The intensities of (Z)-2-penten-1-ol, *cis*-4-heptenal, 2-butanol, 2-propanol, ethyl acetate, isobutyl acetate, butyl 2-methylbutanoate, ammonia-M, ammonia-D, and (E)-2-heptenal increased with air-drying time. The intensities of 3-methyl-1-butanol-M, 1-penten-3-ol-D, 2-methyl-1-propanol-M, 1-propanol-M, 3-pentanone-M, 3-pentanone-D, ethanol-M, ethanol-D, 3-methylbutanal, diethyl acetate, and acetone decreased with air-drying time. The compounds, such as acetoin, 1-pentanol, 3-methyl-1-butanol, heptanal, 1-penten-3-ol, 1-butanol, (E)-2-pentenal, 2-methyl-1-propanol, hexanal, 1-propanol, 1-penten-3-one, 3-pentanone, ethanol, and ammonia, have double peaks, which may be attributable to the presence of monomers and their dimers.

**Table 1:** Results of the qualitative analysis of volatile flavor compounds in hairtails during air-drying.

**No**.	**Compound**	**CAS**	**Formula**	**MW**	**RI**	**Rt/s**	**Dt/RIP relative**	**Peak Intensity (V)**		
								**0 d**	**2 d**	**4 d**
1	Linalool	C78706	C10H18O	154.3	1560.0	1447.682	1.22746	351.08 ± 28.14^b^	346.22 ± 31.97^b^	701.01 ± 93.92^a^
2	Benzaldehyde	C100527	C7H6O	106.1	1550.4	1417.828	1.15722	260.94 ± 34.56^a^	166.57 ± 7.60^b^	194.02 ± 14.08^b^
3	Acetic acid	C64197	C2H4O2	60.1	1504.2	1282.603	1.05758	1658.38 ± 263.86^a^	1471.70 ± 40.31^a^	1652.57 ± 63.34^a^
4	(E,E)-2,4-Heptadiena	C4313035	C7H10O	110.2	1517.2	1319.482	1.19806	198.83 ± 26.83^b^	193.34 ± 33.03^b^	293.97 ± 25.26^a^
5	2-Ethyl-3,5-dimethylpyrazine	C13925070	C8H12N2	136.2	1492.7	1250.992	1.22583	363.86 ± 58.74^a^	221.44 ± 6.46^b^	237.88 ± 33.15^b^
6	1-Octen-3-ol	C3391864	C8H16O	128.2	1483.5	1226.406	1.17192	298.35 ± 19.75^b^	246.92 ± 8.55^c^	472.90 ± 31.86^a^
7	Nonanal	C124196	C9H18O	142.2	1405.3	1034.984	1.47576	416.02 ± 27.02^b^	388.72 ± 42.08^b^	627.83 ± 104.66^a^
8	1-Hexanol	C111273	C6H14O	102.2	1369.6	957.713	1.33201	513.78 ± 49.47^b^	656.92 ± 4.22^b^	1084.94 ± 198.38^a^
9	2,3-Dimethylpyrazine	C5910894	C6H8N2	108.1	1352.9	923.617	1.10564	1782.99 ± 80.62^a^	1057.30 ± 8.22^b^	1927.84 ± 188.34^a^
10	Hexyl propionate	C2445763	C9H18O2	158.2	1352.9	923.617	1.44315	211.65 ± 22.29^b^	93.11 ± 4.02^c^	355.50 ± 77.71^a^
11	Ethyl heptanoate	C106309	C9H18O2	158.2	1340.7	899.508	1.39824	210.99 ± 26.16^a^	110.77 ± 1.70^c^	150.47 ± 10.08^b^
12	(Z)-2-penten-1-ol	C1576950	C5H10O	86.1	1338.7	895.701	0.94485	1144.50 ± 56.80^c^	2390.96 ± 122.51^b^	2911.36 ± 85.86^a^
13	Acetoin-M	C513860	C4H8O2	88.1	1299.2	822.105	1.05929	3653.19 ± 496.27^a^	3614.01 ± 199.78^a^	2108.53 ± 152.02^b^
14	Acetoin-D	C513860	C4H8O2	88.1	1300.6	824.643	1.33306	1584.51 ± 638.96^ab^	2176.59 ± 406.82^a^	814.79 ± 98.52^b^
15	1-Pentanol-M	C71410	C5H12O	88.1	1266.3	767.542	1.25629	1192.17 ± 97.60^a^	1050.28 ± 26.85^ab^	932.59 ± 85.48^b^
16	1-Pentanol-D	C71410	C5H12O	88.1	1266.3	767.542	1.51123	187.49 ± 18.88^a^	159.59 ± 7.41^ab^	140.62 ± 22.95^b^
17	Styrene	C100425	C8H8	104.2	1268.7	771.349	1.05929	175.37 ± 35.29^a^	108.29 ± 3.81^b^	115.43 ± 7.69^b^
18	cis-4-Heptenal	C6728310	C7H12O	112.2	1257.4	753.584	1.15199	216.75 ± 7.37^c^	343.31 ± 19.25^b^	920.82 ± 48.89^a^
19	(E)-2-Hexenal	C6728263	C6H10O	98.1	1233.2	716.786	1.18096	665.18 ± 37.05^a^	379.99 ± 16.22^b^	551.88 ± 106.07^a^
20	3-Methyl-1-butanol-M	C123513	C5H12O	88.1	1222.9	701.559	1.2418	4947.37 ± 130.04^a^	4259.91 ± 61.82^b^	3182.26 ± 352.92^c^
21	3-Methyl-1-butanol-D	C123513	C5H12O	88.1	1223.7	702.828	1.4924	3520.70 ± 309.69^a^	3516.44 ± 97.34^a^	2147.35 ± 718.97^b^
22	Heptanal-M	C111717	C7H14O	114.2	1196.8	664.76	1.33016	1212.59 ± 122.07^a^	479.48 ± 75.33^b^	1269.12 ± 111.89^a^
23	Heptanal-D	C111717	C7H14O	114.2	1198.7	667.298	1.70243	244.06 ± 53.73^b^	68.58 ± 9.06^c^	363.34 ± 53.73^a^
24	1-Penten-3-ol-M	C616251	C5H10O	86.1	1178.5	629.231	0.94485	5712.83 ± 97.17^b^	8230.14 ± 99.90^a^	8101.88 ± 66.23^a^
25	Myrcene	C123353	C10H16	136.2	1177.9	627.962	1.22297	1843.93 ± 54.79^a^	1660.95 ± 13.33^b^	1795.18 ± 93.72^a^
26	1-Penten-3-ol-D	C616251	C5H10O	86.1	1178.5	629.231	1.3461	1355.77 ± 35.79^a^	1077.49 ± 44.47^b^	906.62 ± 25.37^c^
27	2-Hexanol	C626937	C6H14O	102.2	1179.1	630.5	1.5851	1670 ± 76.06^a^	1444.81 ± 22.17^b^	1404.28 ± 83.20^b^
28	1-Butanol-M	C71363	C4H10O	74.1	1164.2	600.046	1.18386	1415.06 ± 142.98^b^	1440.32 ± 44.15^b^	1943.65 ± 203.53^a^
29	1-Butanol-D	C71363	C4H10O	74.1	1163.5	598.777	1.37796	279.41 ± 68.16^b^	322.72 ± 9.87^b^	743.90 ± 174.55^a^
30	(E)-2-Pentenal-M	C1576870	C5H8O	84.1	1150.5	573.399	1.10709	2879.10 ± 171.76^a^	1553.53 ± 14.71^c^	2274.61 ± 96.42^b^
31	(E)-2-Pentenal-D	C1576870	C5H8O	84.1	1151.1	574.668	1.36638	1270.70 ± 76.87^b^	499.71 ± 48.46^c^	2105.72 ± 124.87^a^
32	Isoamyl acetate	C123922	C7H14O2	130.2	1147.1	567.055	1.32437	321.95 ± 111.33^a^	96.70 ± 5.85^b^	229.45 ± 44.52^ab^
33	4-Methyl-3-penten-2-one	C141797	C6H10O	98.1	1129.1	534.063	1.11433	411.70 ± 60.92^a^	226.84 ± 48.09^b^	281.42 ± 75.28^b^
34	2-Methyl-1-propanol-M	C78831	C4H10O	74.1	1111.0	503.072	1.17033	2306.24 ± 91.99^a^	2039.95 ± 5.95^b^	1552.23 ± 62.37^c^
35	2-Methyl-1-propanol-D	C78831	C4H10O	74.1	1109.9	501.108	1.36576	2579.54 ± 348.22^b^	3228.75 ± 139.51^a^	2539.75 ± 196.54^b^
36	Hexanal-M	C66251	C6H12O	100.2	1101.5	487.365	1.26126	3432.09 ± 16.01^a^	2020.17 ± 123.67^b^	1959.56 ± 72.97^b^
37	Hexanal-D	C66251	C6H12O	100.2	1102.1	488.347	1.56527	5855.84 ± 410.08^a^	1596.33 ± 281.56^c^	4513.07 ± 249.48^b^
38	1-Propanol-M	C71238	C3H8O	60.1	1057.0	426.503	1.11333	3828.22 ± 22.86^a^	2350.07 ± 23.60^b^	1645.80 ± 126.99^c^
39	1-Propanol-D	C71238	C3H8O	60.1	1057.8	427.485	1.24769	5442.35 ± 320.05^a^	3636.01 ± 45.99^b^	3787.14 ± 195.96^b^
40	1-Penten-3-one-M	C1629589	C5H8O	84.1	1043.8	410.138	1.07624	524.97 ± 88.64^a^	223.12 ± 45.18^b^	269.16 ± 45.21^b^
41	2-Butanol	C78922	C4H10O	74.1	1039.3	404.606	1.14454	700.65 ± 24.61^c^	2166.71 ± 93.54^b^	2677.04 ± 145.70^a^
42	1-Penten-3-one-D	C1629589	C5H8O	84.1	1043.4	409.585	1.31096	649.45 ± 79.16^a^	126.40 ± 38.66^b^	191.85 ± 29.76^b^
43	3-Pentanone-M	C96220	C5H10O	86.1	1001.7	362.015	1.10853	1713.73 ± 55.15^a^	1041.74 ± 49.14^b^	841.50 ± 29.41^c^
44	3-Pentanone-D	C96220	C5H10O	86.1	1000.7	360.909	1.35442	9029.68 ± 127.01^a^	3294.34 ± 179.87^b^	2288.23 ± 197.76^c^
45	Ethanol-M	C64175	C2H6O	46.1	948.0	319.424	1.04147	5673.04 ± 16.71^a^	3112.39 ± 80.42^b^	2536.57 ± 183.51^c^
46	Ethanol-D	C64175	C2H6O	46.1	950.3	321.084	1.1197	2906.14 ± 216.21^a^	1465.18 ± 28.40^b^	1071.27 ± 64.97^c^
47	3-Methylbutanal	C590863	C5H10O	86.1	931.6	307.809	1.40286	8706.88 ± 65.60^a^	2412.22 ± 98.11^b^	1535.22 ± 151.75^c^
48	2-Butanone	C78933	C4H8O	72.1	916.9	297.852	1.24638	3961.07 ± 133.02^a^	847.31 ± 72.57^b^	702.58 ± 103.10^b^
49	Butanal	C123728	C4H8O	72.1	893.2	282.365	1.28115	2805.43 ± 321.54^a^	286.84 ± 18.30^b^	464.75 ± 22.29^b^
50	Diethyl acetal	C105577	C6H14O2	118.2	908.6	292.321	1.0278	1264.41 ± 23.89^a^	600.65 ± 38.62^b^	430.46 ± 57.82^c^
51	Acetone	C67641	C3H6O	58.1	843.6	252.495	1.11598	5133.46 ± 138.92^a^	3651.21 ± 360.38^b^	1296.95 ± 69.69^c^
52	2-Methylpropanal	C78842	C4H8O	72.1	836.8	248.624	1.28736	1030.03 ± 102.43^a^	62.38 ± 3.51^b^	17.02 ± 1.49^b^
53	Dimethyl sulfide	C75183	C2H6S	62.1	797.5	227.605	0.95577	428.55 ± 26.09^b^	789.07 ± 74.54^a^	79.33 ± 34.09^b^
54	Propanal	C123386	C3H6O	58.1	819.6	239.22	1.12343	9865.14 ± 110.95^a^	9300.30 ± 57.07^b^	9192.91 ± 20.52^b^
55	Acetaldehyde	C75070	C2H4O	44.1	765.1	211.564	0.97937	3582.32 ± 40.85^a^	3554.77 ± 1.04^a^	2697.59 ± 328.66^b^
56	Trimethylamine	C75503	C3H9N	59.1	845.6	253.602	1.14951	27490.84 ± 167.30^c^	66294.21 ± 1646.89^b^	100364.74 ± 3676.58^a^
57	2-Propanol	C67630	C3H8O	60.1	918.6	298.958	1.20415	614.69 ± 79.93^c^	2296.09 ± 66.75^b^	2493.07 ± 90.62^a^
58	Ethyl Acetate	C141786	C4H8O2	88.1	901.9	287.896	1.33704	59.20 ± 4.40^c^	100.94 ± 0.19^b^	131.72 ± 18.98^a^
59	Toluene	C108883	C7H8	92.1	1065.4	437.241	1.04271	963.45 ± 35.73^a^	531.48 ± 24.28^c^	459.43 ± 44.11^b^
60	2-Ethylfuran	C3208160	C6H8O	96.1	974.1	338.784	1.31344	51.92 ± 2.76^b^	89.95 ± 11.59^b^	283.69 ± 31.08^a^
61	Ethyl butanoate	C105544	C6H12O2	116.2	1064.1	435.582	1.20788	448.54 ± 31.92^b^	526.10 ± 38.28^b^	1204.13 ± 56.56^a^
62	Isobutyl acetate	C110190	C6H12O2	116.2	1042.9	409.031	1.23893	135.04 ± 8.12^c^	438.27 ± 125.94^b^	905.71 ± 125.90^a^
63	Butyl 2-methylbutanoate	C15706737	C9H18O2	158.2	1231.8	714.708	1.3726	104.25 ± 8.83^c^	260.06 ± 9.91^b^	1204.85 ± 130.24^a^
64	3-Ethylpyridine	C536787	C7H9N	107.2	1369.4	957.426	1.10929	297.58 ± 18.57^ab^	260.68 ± 0.29^b^	328.90 ± 31.94^a^
65	Propanoic acid	C79094	C3H6O2	74.1	1639.0	1718.563	1.11064	341.96 ± 26.45^b^	355.86 ± 29.40^b^	475.32 ± 19.29^a^
66	Ammonia-M	C7664417	H3N	17.0	1247.4	738.072	0.85103	11173.06 ± 297.69^c^	15343.04 ± 459.22^b^	37416.07 ± 3460.77^a^
67	Ammonia-D	C7664417	H3N	17.0	1248.5	739.754	0.89785	44098.91 ± 484.22^c^	50368.44 ± 721.11^b^	62275.17 ± 785.19^a^
68	Terpinen-4-ol	C562743	C10H18O	154.3	1634.5	1701.744	1.22129	326.94 ± 72.41^a^	289.84 ± 33.20^a^	361.58 ± 20.79^a^
69	(E)-2-Octenal	C2548870	C8H14O	126.2	1438.9	1113.114	1.3362	158.26 ± 5.39^a^	123.94 ± 8.81^b^	182.25 ± 24.35^a^
70	2-Cyclohexen-1-one	C930687	C6H8O	96.1	1413.2	1052.791	1.12247	88.53 ± 12.30^b^	74.53 ± 2.52^b^	118.60 ± 9.77^a^
71	(E)-2-Heptenal	C18829555	C7H12O	112.2	1328.6	876.136	1.23757	39.17 ± 2.95^c^	55.10 ± 1.36^b^	142.47 ± 12.46^a^
72	Octanal	C124130	C8H16O	128.2	1297.0	818.157	1.4087	211.50 ± 6.26^a^	120.50 ± 13.63^b^	152.60 ± 24.13^b^
73	beta-Pinene	C127913	C10H16	136.2	1152.0	576.274	1.21788	255.93 ± 20.53^b^	229.35 ± 3.71^b^	342.10 ± 27.37^a^
74	Acrolein	C107028	C3H4O	56.1	868.6	267.113	1.05761	587.96 ± 125.62^a^	29.33 ± 0.86^b^	18.33 ± 2.32^b^
75	2-Heptanone	C110430	C7H14O	114.2	1193.0	659.507	1.2648	145.10 ± 7.12^b^	1471.70 ± 40.31^b^	222.15 ± 15.75^a^

Different lowercase letters denote significant differences (*P* < 0.05) among the same substances at different air-drying durations.

### 3.4. Fingerprints and heatmap of VOCs in hairtails during air-drying

**Figure 4:**
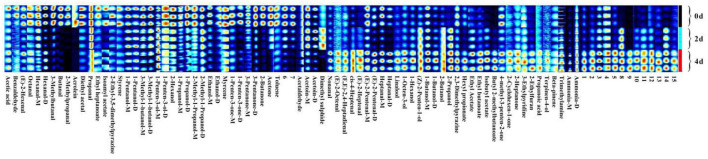
Fingerprint of volatile substances in hairtails.

To visualize changes in VOCs during the air-drying processes of hairtails, the VOCs were analyzed qualitatively (Figure 4). Each row represents all signal peaks of a sample, and each column represents a single volatile compound of different samples. Its color shows the strength of signal peaks in the hairtail samples, and the brighter the color, the greater the strength. Figure 4 shows that the VOCs were similar among various air-dried hairtail samples, but the brightness of each spot showed obvious changes, and the peak intensity was relatively different, indicating that the content of VOCs during the air-drying process was distinct among hairtail samples. The content of acetic acid, benzaldehyde, (E)-2-hexenal, octanal, hexanal, 3-methylbutanal, butanal, 2-methylpropanal, acrolein, diethyl acetate, ethyl heptanoate, isoamyl acetate, 2-ethyl-3,5-dimethylpyrazine, styrene, 3-methyl-1-butanol-M, 1-penten-3-ol-D, 2-methyl-1-propanol-M, hexanal-D, 1-propanol-M, 3-pentanone, ethanol, acetone, toluene, and 28 other VOCs in the 0-day hairtail samples was higher than those of the other two samples, indicating that the mentioned compounds could turn out to be important VOCs in fresh hairtails. The content of most of the VOCs was lowest in the 2-day hairtail samples, with only acetoin and dimethyl sulfide markedly higher than the other two groups, indicating that these compounds play a crucial role in the flavor of the 2-day hairtail samples. The content of nonanal, (E)-2-octenal, (E,E)-2,4-heptadienal, *cis*-4-heptenal, (E)-2-heptenal, linalool, 1-octen-3-ol, 1-hexanol, (Z)-2-penten-1-ol, 1-butanol-D, 2-butanol, 2-propanol, ethyl acetate, ethyl butanoate, isobutyl acetate, butyl 2-methylbutanoate, trimethylamine, ammonia, and 30 other VOCs in the 4-day hairtail samples increased with air-drying time.

According to the results of GC-IMS, alcohols are the first largest VOCs' component in hairtails. Alcohols are mainly formed by lipid oxidation, Maillard reaction, and reduction of the carbonyl compound. Moreover, the threshold of alcohols is very high and contributes less to the formation of overall flavor; these compounds include ethanol, 1-butanol, 1-propanol, and other short straight-chain alcohols. When the concentration of ethanol is higher, it generates herb, xylon, and fat odor (7). The content of ethanol decreased during the air-drying process, as ethanol is a highly volatile compound and is a part of the ethanol participate esterification reaction to form flavor. A previous study suggested that ethanol could be used as a substrate for ester synthesis in fish products (27). Some long straight-chain alcohols have relatively low threshold values and significantly impact hairtail flavors such as 1-pentanol, 1-hexanol, 1-octen-3-ol, and 3-methyl-1-butanol (7). The Strecker degradation of leucine or isoleucine leads to the formation of 3-methyl-1-butanol, which imparts a malty aroma sensation that contributes to the overall aroma of hairtail (28). The oxidation of oleic acid produces 1-pentanol, which also contributes to the green and woody aroma (29). Both compounds accumulated mainly in the 0-day hairtail. Alcohols, such as 1-hexanol, has a rosin and flower aroma, and 1-octen-3-ol has a mushroom aroma, and all these aromas are mainly present in the 4-day hairtail samples. Compounds 1-hexanol and 1-octen-3-ol are formed from the oxidation of oleic acid (C18:1n9c) or palmitoleic acid (C16:1) (30) and C18:3n3 or C18:2n6 (31). A previous study showed that the content of 1-octen-3-ol is highly correlated to TBARS values and thus could reflect the oxidation levels of fat in fish (32). The signal peak of 1-octen-3-ol was enhanced with air-drying time, indicating that the level of lipid oxidation in hairtail gradually increased during the air-drying process, which confirmed our earlier results.

Aldehydes are important VOCs in meat and meat products that present with a smell of fat, green, paint, faint scent, and fruit and are the second largest VOC component in hairtails (33, 34). Aldehydes are generated from the oxidative degradation of unsaturated fatty acids and the Strecker degradation reaction of amino acids (35). Due to the low odor threshold, these aldehydes contribute largely to the flavor of food (11). Previous studies detected nonanal, hexanal, octanal, and heptanal in salted dry fish products and were verified to be important VOCs (36, 37), which agreed with the results of the current study. Nonanal and octanal were produced by the oxidation of oleic acid. Nonanal has a gassy and fatty odor, while octanal generates a fruity and fatty taste (15). The oxidation of linoleic acid and arachidonic acid produces hexanal (fragrance of apple, leaf, and delicate) and heptanal (flavor of nutty and fruity green) (38, 39), respectively. The signal peaks of hexanal and octanal were higher in the 0-day hairtail samples, and the signal peaks of nonanal and heptanal were higher in the 4-day hairtail samples, giving the hairtails their distinctive flavor. Benzaldehyde (flavor of almond bitterness) is formed from the Strecker degradation of phenylalanine and highly accumulated in 0 d hairtails (40). Another compound with a low odor threshold, 3-methylbutanal, gives the hairtail a fruity and cheese aroma (41), which is formed by the Strecker degradation of isoleucine or leucine and which largely contributes to fish odor.

Ketones are mainly produced from lipid oxidation, Maillard reaction, and amino acid degradation (42). Furthermore, ketones are also generated by the oxidation of alcohols and the decomposition of esters (43). These ketones are usually related to creamy, spicy, fatty, and fruity flavors (11, 44), which are also important for the generation of aroma in hairtails. The levels of 2-heptanone significantly increased with air-drying time. 2-Heptanone has a blue cheese odor, which is obtained through the decomposition of linoleic acid (11). Acetoin has a buttery aroma and the strongest signal peak on day 2 of air-drying (45). Acetoin is a precursor of diacetyl and the by-product of the metabolism of certain microbial carbohydrates, which play crucial roles in the synthesis of diols, esters, and glycosides and which have a significant impact on the overall aroma of food (46). Acetone, 2-butanone, 3-pentanone, and 1-penten-3-one are the most abundant VOCs in the 0-day hairtail samples. Acetone imparts a buttery smell to hairtails (47). 2-Butanone is related to the taste of milk (48) and is a spoilage marker for freshness in fish (49). Due to the influence of the smell of freshwater microalga, 3-pentanone has a wispy and fruity flavor (50). 3-Pentanone is highly enriched in the 0-day hairtail samples, which agreed with the findings on hairtails by Ding et al. (8). 1-Penten-3-one has a spicy and garlic odor. The level of ketones significantly differed among the three samples and contributed to a major effect on hairtail aroma.

Esters are important VOCs that impart a pleasant odor (fruit smell) to food (51). Ester compounds are synthesized by the esterification of alcohols and carboxylic acids and are also derived from the alcoholysis of acylglycerols and fatty alcohols (52). Moreover, short-chain esters may possess a sweet or fruity odor, while long-chain esters produce fatty aromas (53). Ethyl acetate has a pleasant, fruity, and brandy-like aroma (40, 54) and was observed at its highest level on day 4. Ethyl heptanoate, a compound with a fruity and winy odor (40), reached its highest level on day 0. The formation of esters in dry-cured meat is correlated to specific microbial enzymes (55). The levels of several esters increased with air-drying time and may be caused by the Maillard reaction in hairtails during the air-drying process, providing a unique flavor in hairtails. Subsequently, compounds formed from ester degradation consistently react with the intermediate product of amino acids, contributing to all aromatic odors in meat. These studies indicate that air-drying effectively increases the concentration of esters in processed hairtails compared with raw hairtails.

Pyrazines are special flavor substances of the Maillard reaction and have a peculiar baking and nutty taste (56, 57). With its high threshold value, acids are mainly formed from the hydrolysis of fats and do not contribute to the flavor of meat (58). 2-Ethylfuran is the degradation product of thiamine and has a stronger burnt flavor, which is mainly enriched on day 4. Dimethyl sulfide generates synergistic effects with other flavors, and its content is high on day 2 and may have impacted fruity odor expression (59). Trimethylamine is the product of facultative anaerobe reduction of trimethylamine oxide, which is the main component of fishy odor. The level of trimethylamine gradually increased during the air-drying process, possibly because trimethylamine oxide is extremely unstable and can be reduced easily to form trimethylamine by the action of the enzyme. The signal peak of ammonia was enhanced during the air-drying process, possibly due to the decomposition of proteins by microorganisms, transformation to peptides and amino acids, and the formation of ammonia. Trimethylamine and ammonia emit a bad smell and are considered putrefaction markers of flavor in hairtails.

### 3.5. Principal component analysis (PCA) and correlation analysis of flavor compounds in hairtails during the air-drying process

**Figure 5:**
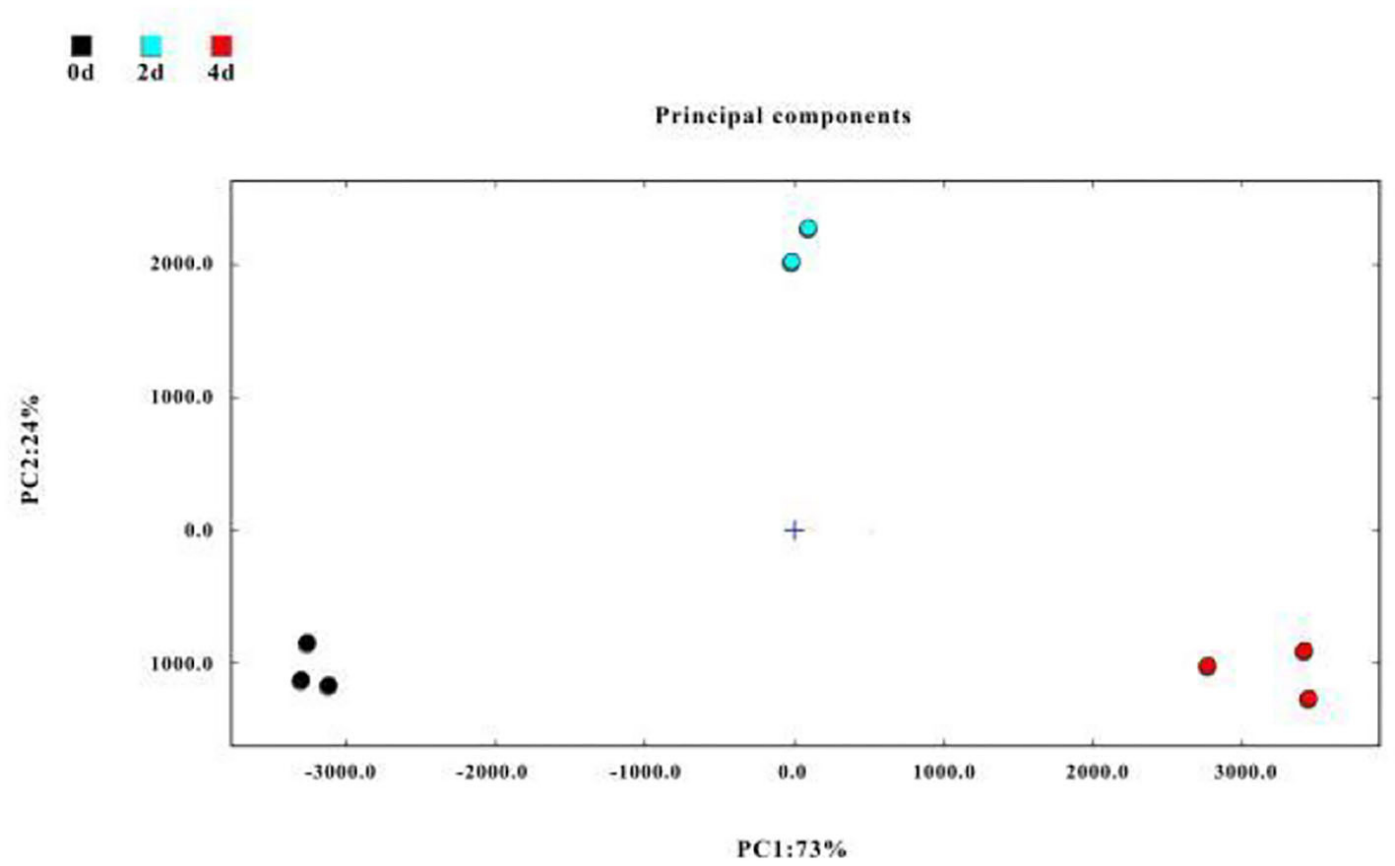
Principal component analysis (PCA) of flavor compounds in hairtails during the air-drying process.

Principal component analysis is a multivariate statistical analysis tool that uses a multivariate linear transformation to extract information on significant variables (60). The PCA model is selected as the separation model when the cumulative contribution rate reaches 60%. In this study, PCA was used to analyze changes in identified VOCs in air-dried hairtail samples (Figure 5), in which the cumulative variance contribution rate of the first part PC1 (first principal component) (73%) and the second part PC2 (second principal component) (24%) was 97%. The 0-day, 2-day, and 4-day groups were located in different places and far away from each other; the hairtail samples were clearly distinguished, indicating the relationship of flavor compounds among different air-drying times.

**Figure 6:**
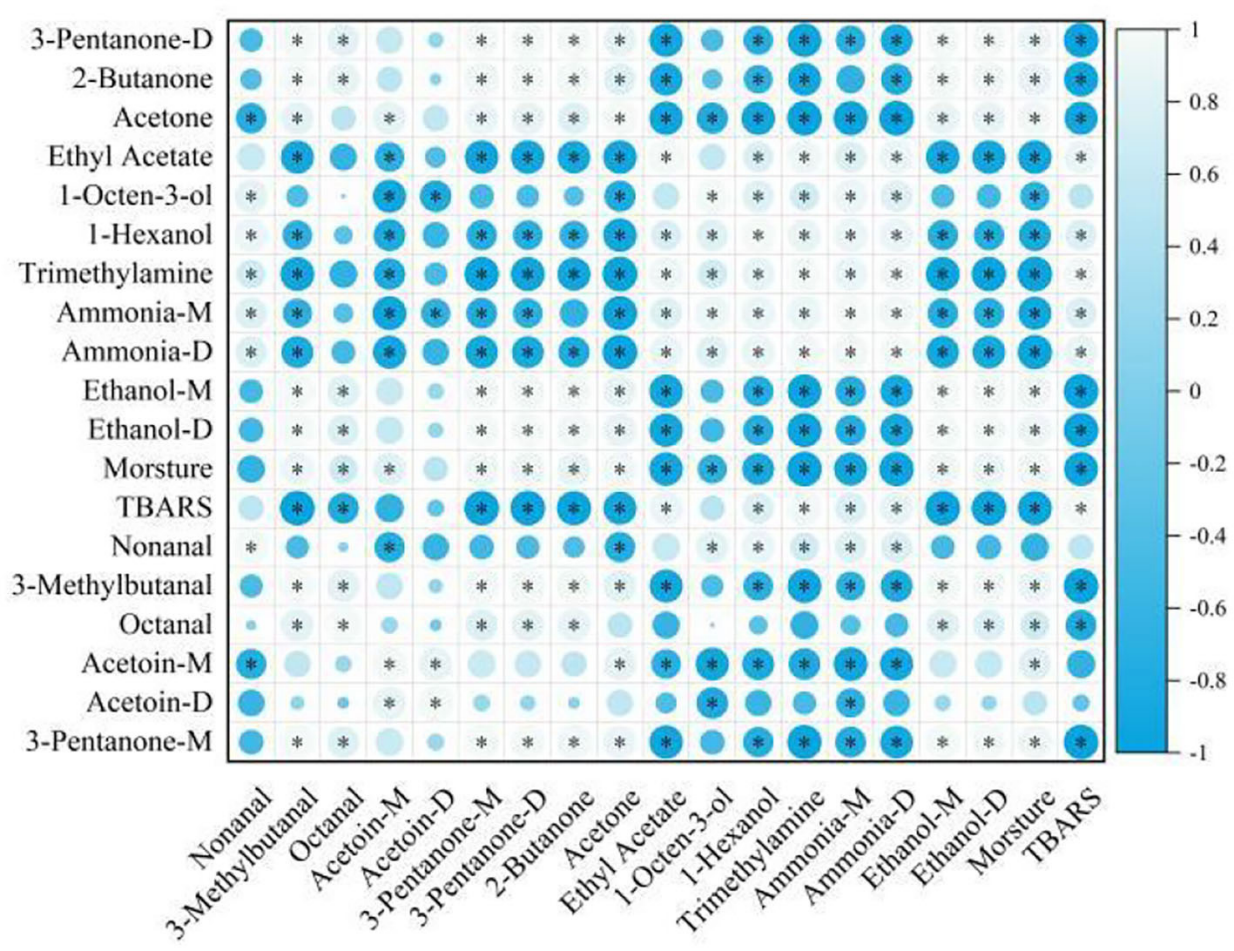
Correlation analysis of flavor compounds in hairtails during the air-drying process.

Moreover, a correlation analysis (Figure 6) was performed in the present study to determine the relationship between 15 VOCs using Spearman's correlation tests. The moisture content and TBARS values were significantly correlated (*P* < 0.05). This result was consistent with the experimental conclusions of this study. Ethanol showed a significantly negative correlation with ammonia, trimethylamine, 1-hexanol, and ethyl acetate (*P* < 0.05). The alcohols and esters were significantly negatively correlated with acetoin (*P* < 0.05) but were significantly positively correlated with aldehydes. This may be the reason why lipid oxidation, the degradation of proteins, and the growth of microorganisms lead to changes in VOCs during the air-drying process, with these VOCs mutually transformed and emitting a distinctive flavor in air-dried hairtails.

## 4. Conclusions

In this study, volatile compounds of hairtails during air-drying were analyzed using HS-GC-IMS. The physicochemical results revealed a decrease in moisture content, while the TBARS values increased with air-drying time. A total of 75 VOCs, including 22 alcohols, 21 aldehydes, 11 ketones, 7 esters, 4 hydrocarbons, 3 nitrogen-bearing, 2 pyrazines, 2 acids, 1 furan, 1 sulfur-bearing, and 1 pyridine, were identified in hairtails. Among these, ethanol, 3-methyl-1-butanol, 1-pentanol, hexanal, octanal, benzaldehyde, and 3-methylbutanal were the main volatile components in the 0-day hairtails. Acetoin and dimethyl sulfide were the characteristic VOCs in 2 d hairtails. Compounds, such as 1-hexanol, 1-octen-3-ol, nonanal, heptanal, 2-heptanone, ethyl acetate, trimethylamine, and ammonia, were identified in the 4-day hairtail samples and thus considered biomarkers. In addition, our results showed that different samples were relatively independent and distinguishable in the PCA chart. Correlation analysis of flavor compounds indicated that these compounds were significantly correlated and promoted the flavor of hairtails *via* a synergistic effect. As an emerging technology, HS-GC-IMS could be a rapid and reliable method for analyzing flavor characteristics, which could readily distinguish different samples of hairtails during the air-drying process.

## Data availability statement

The original contributions presented in the study are included in the article/supplementary material, further inquiries can be directed to the corresponding authors.

## Ethics statement

No animals or humans were used as subjects in this study.

## Author contributions

YL, YD, YW, QD, JX, and JJ contributed to the conception and design of the opinion-study and drafted the manuscript first. SB, YH, HL, and BZ edited, revised, and approved the final version of the submitted manuscript. All authors contributed to the article and approved the submitted version.
